# Colonization of Dogs and Their Owners with *Staphylococcus aureus* and *Staphylococcus pseudintermedius* in Households, Veterinary Practices, and Healthcare Facilities

**DOI:** 10.3390/microorganisms10040677

**Published:** 2022-03-22

**Authors:** Christiane Cuny, Franziska Layer-Nicolaou, Robert Weber, Robin Köck, Wolfgang Witte

**Affiliations:** 1 Robert Koch Institute, 38855 Wernigerode, Germany; layerf@rki.de; 2 Helmholtz Centre for Infection Research, 38124 Braunschweig, Germany; robert.weber@helmholtz-hzi.de; 3 Institute of Hygiene, University Hospital, 48149 Münster, Germany; r.koeck@drk-kliniken-berlin.de; 4 Institute of Hygiene, DRK-Kliniken, 13359 Berlin, Germany; 5 Braunlagerstr. 7, 38875 Elend, Germany; ewwitte@t-online.de

**Keywords:** zoonosis, transmission of *Staphylococcus aureus*, household contacts, dogs, humans

## Abstract

There are uncertainties with respect to the transmission of methicillin-susceptible and methicillin-resistant *Staphylococcus aureus* (MSSA and MRSA) and *Staphylococcus pseudintermedius* between dogs and humans. In this study, we investigated concomitant nasal colonization of dogs and humans in three cohorts. Cohort I, households owning dogs: In 42 of 84 households, 66 humans (36.9%) and 10 dogs (8.9%) carried *S. aureus*. MRSA, attributed to sequence type (ST) 22 and ST130, were detected in two (1.1%) of the humans but in none of the dogs. Typing by means of *spa*-typing and whole-genome sequencing (WGS) indicated eight transmissions of *S. aureus* between humans and dogs in 8 of 42 (19.0%) households with human *S. aureus* carriers, whereas in 11 of 38 (29.0%) households with ≥two persons and *S. aureus* colonization of humans, 15 human-to-human transmissions were observed (*p* = 0.43). *S. pseudintermedius* was isolated from 42 dogs (37.5%), but from only one human (0.6%). In this case, WGS-based typing indicated strong relatedness of this isolate with a canine isolate from the same household. Cohort II, dogs and their owners visiting a veterinary practice: Among 17 humans and 17 dogs attending a veterinary practice, MSSA was detected in three humans and two dogs, and *S. pseudintermedius* in only six dogs. Cohort III, dogs used for animal-assisted interventions in human healthcare facilities and their owners: MSSA was obtained in 1 of 59 dogs (1.7%) and in 17 of 60 (28.3%) of the dog owners, while *S. pseudintermedius* was isolated from seven (12%) dogs and one (1.7%) human owner. We conclude that the risk of exchanging *S. aureus*/MRSA between humans and dogs is higher than that for *S. pseudintermedius*.

## 1. Introduction

*Staphylococcus aureus* (*S. aureus*) is widely disseminated as a colonizer and as an opportunistic pathogen among humans and other animal species. In the human community, it colonizes the anterior nares of about 25–35% of healthy persons [1,2]. The population structure of *S. aureus* consists of several clonal complexes, some of which are associated with defined animal species, while others are less host-specific [3]. Methicillin-resistant *S. aureus* (MRSA) is globally prevalent in nosocomial settings as healthcare-associated MRSA (HA-MRSA), which is mainly due to the spread of epidemic clonal lineages [4]. In addition, MRSA emerges in the community without any relation to the healthcare facilities (CA-MRSA, [5]. HA-MRSAs, especially those attributed to lineage ST22, were also observed as nosocomial pathogens in veterinary hospitals [6]. Livestock-associated MRSA (LA-MRSA), attributed to clonal complex (CC) 398 as defined by multilocus sequence typing (MLST), originally emerged in livestock before a particular subpopulation became increasingly prevalent in animal hospitals during the past decade [7,8,9].

*Staphylococcus pseudintermedius* is a member of the *Staphylococcus intermedius* group (SIG), besides *Staphylococcus intermedius* and *Staphylococcus delphini* [10]. These three coagulase-positive staphylococcal species are commensals of the skin and mucous membranes. *Staphylococcus pseudintermedius* is the major coagulase-positive staphylococcal species that colonizes dogs and cats, and also the most prevalent causative agent of canine bacterial infections [11]. Methicillin-resistant *S. pseudintermedius* (MRSP) exhibiting multiple resistance phenotypes has globally emerged as nosocomial pathogens in hospitals for small animals [12]. Although still infrequent, *S. pseudintermedius* was also isolated from infections in humans after dog bites [13,14], as well as from other kinds of infections, including septicemia [15]. So far, studies on human nasal colonization with *S. pseudintermedius* in dog-owning households were reported in Korea [16] and Portugal [17], where a prevalence of 3–4.5% was found in humans and 25–65.9% in dogs, respectively.

In 2020, 10.7 million dogs were living in 21% of German households [18]. Dogs and cats are increasingly regarded as family members. Therefore, the question about mutual transmission of pathogens is of particular interest. In households, staphylococci can be transmitted either through direct contact during owner-pet interactions or through secondary contact with contaminated surfaces [19]. Previous studies suggested that *S. aureus* can be transmitted between dogs and their human owners, as isolates exhibited identical phage patterns [20]. Although considerably less frequent than in humans, *S. aureus* was described as a colonizer of dogs [21]. The question of whether this colonization is associated with *S. aureus* carriage by human contact persons has been addressed by a few previous studies in Korea [16], Hong Kong [22], and the United States [23], which resulted in different findings with respect to the frequencies of concomitant colonization of dogs and owners. This could be due to the study design with respect to concomitant sampling of humans and dogs, and to not sampling all household members. Intrahousehold transmission of MRSA from colonized or infected humans to dogs was observed in North America [24,25]. The introduction of human HA-MRSA strains into veterinary hospitals by dogs having acquired them from their owners was reported for MRSA ST22 in Europe [26] and ST5 (“USA100”) in North America [27,28].

Animal-assisted therapeutic interventions (AAI) are, meanwhile, established in human healthcare facilities, such as nursing homes, rehabilitation care centers, and hospitals, in many countries. The aim of AAI is to improve the patients’ emotional, cognitive, or neurological functioning [29]. Although the results on the effectiveness of AAI are altogether heterogeneous [29], it seems to be particularly effective in children and adults suffering from post-traumatic stress disorder symptoms [30]. In this context, the possibility of transmitting of HA-MRSA by dogs used for AAI is of particular interest. So far, this was reported from Canada [31] and the UK [32]. 

This study aims to answer questions about the emergence of both *S. aureus* and *S. pseudintermedius* in humans and dogs, and to analyze the transmission. We sampled humans and dogs differentiated into three cohorts: (I) as household contacts, (II) as visitors at veterinary practices, and (III) in human healthcare settings. 

## 2. Materials and Methods

Three different cohorts of participants were enrolled: households owning dogs (cohort I), dogs and their owners visiting a veterinary practice (cohort II), and dogs used for AAI in human healthcare facilities and their owners (cohort III). The study was performed from 2019–2020.

### 2.1. Study Populations and Recruitment

Cohort I: The study was performed in the Landkreis Harz region in Central Germany (214,446 inhabitants, 2104.54 km^2^). There are three primary care hospitals in this area. A total of 83 households were enrolled. Recruitment was based on directly contacting several families owning dogs in five villages and three towns. In a second step, households already participating in the study referred to other dog-owning households. A prerequisite for participation was the agreement of the contacted households, documented by the return of a declaration of consent. The participants were provided with swabs, as well as an instruction for swab-self-collection for humans and dogs, and a short questionnaire asking for information on basic demographic characteristics, such as age, sex, and potential risk factors for colonization with *S. aureus* (e.g., antibiotic treatment, hospital stay during the six months prior to sampling). The feasibility of self-collection of nasal swabs was previously reported [33]. Households in which dogs with *S. aureus* carriage were detected were asked to provide a second nasal sample from the dogs and owners 12 months after the initial sampling.

Cohort II: 17 dogs and their owners were randomly selected by a veterinarian and instructed with respect to self-sampling and data provision as in cohort I.

Cohort III: Supported by the organization “Animals as Therapy” (www.tierealstherapie.at/tat-waz/, accessed on 21 February 2022) the owners of dogs used for AAI in human regional healthcare facilities were directly contacted. The further procedure was as described for cohort I.

The study was approved by the ethical committee of the medical faculty of the University of Magdeburg (#33/14).

### 2.2. Microbiological Analysis

The eSwab^TM^ system (MAST Diagnostics, Reinfeld, Germany) was used for taking swabs from both nostrils of humans and dogs. Aliquots were streaked on CHROMagar^TM^ MRSA (MAST) and, in parallel, on Mueller–Hinton blood agar plates (Oxoid, Wesel, Germany). After incubation at 37 °C for 18–24 h, one suspicious colony was subcultured on sheep blood agar (except in the case of differences in colonial morphology and hemolysis). Confirmation of *S. aureus* was performed by demonstration of the clumping factor and, additionally, by the tube coagulase test. For proof of the clumping factor, we used a solution of fibrinogen from human plasma (Sigma-Aldrich, Taufkirchen, Germany) of 2 mg/mL 0.85% NaCl. For the tube coagulase test, fresh ready-to-use human plasma (DRK blood donation service, Springe, Germany) was used. In the case of negative results, we performed a PCR for the region of *tuf* that is specific for *S. aureus* using the primers and PCR conditions as described [34]. For the identification of *S. pseudintermedius*, presumptive colonies were subcultured on blood agar plates and subjected to PCR analysis according to [35]. For PCR analysis, genomic DNA was extracted using the DNeasy tissue kit (Qiagen, Hilden, Germany), and lysostaphin (100 mg/L; Sigma, Taufkirchen, Germany) for bacterial lysis. 

*Antimicrobial susceptibility testing*: The following antibiotics were tested: penicillin (PEN), oxacillin (OXA), fosfomycin (FOS), gentamicin (GEN), linezolid (LIN), erythromycin (ERY), clindamycin (CLI), tetracycline (TET), tigecycline (TIG), vancomycin (VAN), teicoplanin (TEI), ciprofloxacin (CIP), trimethoprim/sulfamethoxazole (TRS), fusidic acid–sodium (FUS), rifampicin (RIF), mupirocin (MUP), cefoxitin (CEF), moxifloxacin (MOX), and daptomycin (DAP). The testing was performed using a broth microdilution and applying EUCAST clinical breakpoints for humans (version 9.0, valid from 1 January 2019).

### 2.3. Molecular Characterization

DNA extraction: Strains were grown overnight in tryptic soy broth at 37 °C. For PCR analysis, genomic DNA was extracted using the DNeasy tissue kit (Qiagen, Hilden, Germany), and lysostaphin (100 mg/L; Sigma, Taufkirchen, Germany) for bacterial lysis.

*PCR for resistance genes:* PCR analysis was performed for *mec*A and *mec*C in order to confirm MRSA as previously described [36]. 

*Spa-typing, BURP and MLST:* All *S. aureus* isolates were subjected to *spa*-typing as previously described [36]. Related *spa*-types (costs ≤ 4) were grouped into *spa*-clonal complexes (*spa*-CC) by use of the BURP algorithm (Ridom StaphType software version 2.2.1, Ridom, Münster, Germany). The *spa*-CCs were allocated to MLST CCs (Ridom SpaServer—*Spa*-types; database of the German National Reference Center for Staphylococci and Enterococci). Subsets of the isolates were subjected to MLST as described elsewhere [36].

### 2.4. Whole-Genome Sequencing and Phylogenetic Analyses

Whole-genome sequencing: DNA quantification was carried out using the Qubit dsDNA HS Assay Kit (Invitrogen/Thermo Fisher Scientific, Karlsruhe, Germany). A total of 1 ng of extracted DNA was employed for library preparation using the Nextera XT DNA Library Prep Kit according to the manufacturer’s instructions (Illumina, San Diego, CA, USA). Sequencing was performed on an Illumina MiSeq platform in paired-end mode with a final readout of 2 × 250 bp. The quality of the raw read data was assessed by an in-house-developed pipeline (Qcumber-2). Genome assembly and core-genome multilocus sequence typing (cgMLST): The raw read files were imported into Ridom SeqSphere+ version 8.1.0 (Ridom GmbH, Münster, Germany (https://www.ridom.de/seqshpere/ug/v81/Version_History.html, accessed on 21 February 2022) and assembled de novo using SPAdes (version 3.13.1 (https://www.ncbi.nlm.nih.gov/pmc/articles/PMC3342519/; accessed on 21 February 2022), default settings, integrated in the Linux version of SeqSphere). For the *S. aureus* isolates, the *S. aureus* cgMLST scheme version 1.3 (http://pubmed.ncbi.nlm.nih.gov/24759713/; accessed on 21 February 2022), implemented into SeqSphere+, was applied. For *S. pseudintermedius*, an ad hoc cgMLST was established according to Ridom SeqSphere guidelines, with *S. pseudintermedius* ED99 as a seed genome (GenBank accession number CP002478). Minimum spanning trees were calculated on the basis of cgMLST by ignoring pairwise missing values. To detect closely related *S. aureus* and *S. pseudintermedius* isolates, the threshold for cluster distance determination was set to 10 and 15 cgMLST alleles, respectively.

## 3. Results

This study included 179, 17, and 60 humans in cohorts I, II, and III, respectively. Moreover, 112, 17, and 59 dogs were included in the three cohorts.

### 3.1. Cohort I: Humans and Dogs in Household Contacts

Overall, 179 persons participated in the study, representing all members of 84 households. Among these households, 18 were single households (21.4%), 48 comprised two persons (45.2%), and 18 comprised ≥ three persons. In the 84 households, there were 112 dogs, which were included in the study (one dog in 61 households, two dogs in 19 households, ≥three dogs in 4 households).

#### 3.1.1. *S. aureus* Nasal Carriage

*S. aureus* was detected in 66/179 human participants (36.9%); 64 (35.8%) carried methicillin-susceptible *S. aureus* (MSSA), and 2 carried MRSA (1.1%). *S. aureus* nasal carriage was significantly more frequent among persons suffering from atopic eczema (Table 1). For the other variables, an association with *S. aureus* carriage was not apparent. For all 112 dogs living in these households, the overall distribution of risk factors for *S. aureus* carriage was as follows: a previous stay in an animal hospital was reported for 8 (6.6%), a previous antibiotic prescription for 25 (21.5%), skin lesions for 6 (5.4%), and bite injuries for 1 (0.9%). Among these 112 dogs, 10 (7.8%) carried *S. aureus* (all of which were MSSA). Of these, two were previously treated with an antibiotic, one was treated in a veterinary hospital, and one had a skin lesion. 

Typing attributed the *S. aureus* isolates of humans to MLST clonal complexes CC1 (3), CC5 (1), CC7 (4), CC8 (9), CC12 (2), CC15 (12), CC22 (5), CC30 (10), CC45 (15), CC97 (1), CC398 (1), CC130 (1), and ST133 (3). The two MRSA isolates were associated with CC22 (harboring *mec*A; phenotypically resistant to penicillin, cefoxitin, oxacillin, erythromycin, clindamycin, ciprofloxacin, and moxifloxacin) and CC130 (harboring *mec*C; phenotypically resistant to penicillin, cefoxitin, and oxacillin only), respectively.

#### 3.1.2. *S. aureus* Intrahousehold Transmission

Human *S. aureus* carriage was found in 42 (50.0%) of the 84 households. There were 18 single households with four persons carrying *S. aureus*. More than one person lived in 66 households (Table 2). Overall, human *S.aureus* carriage affected 4/18 (22.2%) single households vs. 38/66 (57.6%) non-single households (*p* = 0.015); or related to the number of persons, 4/18 (22.2%) persons in single households vs. 62/161 (38.5%) persons in non-single households (*p* = 0.20643). In 17 of the 66 non-single households, more than one person was concomitantly colonized. The observation of identical *spa*-types and resistance phenotypes in isolates from different individuals from the same household would initially indicate intrahousehold transmission. This was the case for 11 of these 17 households (Supplemental Table S1). Here, we observed 15 transmissions (calculated as the number of humans carrying *S. aureus* with matching typing characteristics minus one). Based on the 38 non-single households with at least one *S. aureus* carrier, where transmission could actually happen, the intrahousehold, human-to-human transmission rate was 11/38 (29.0%).

**Figure 1:**
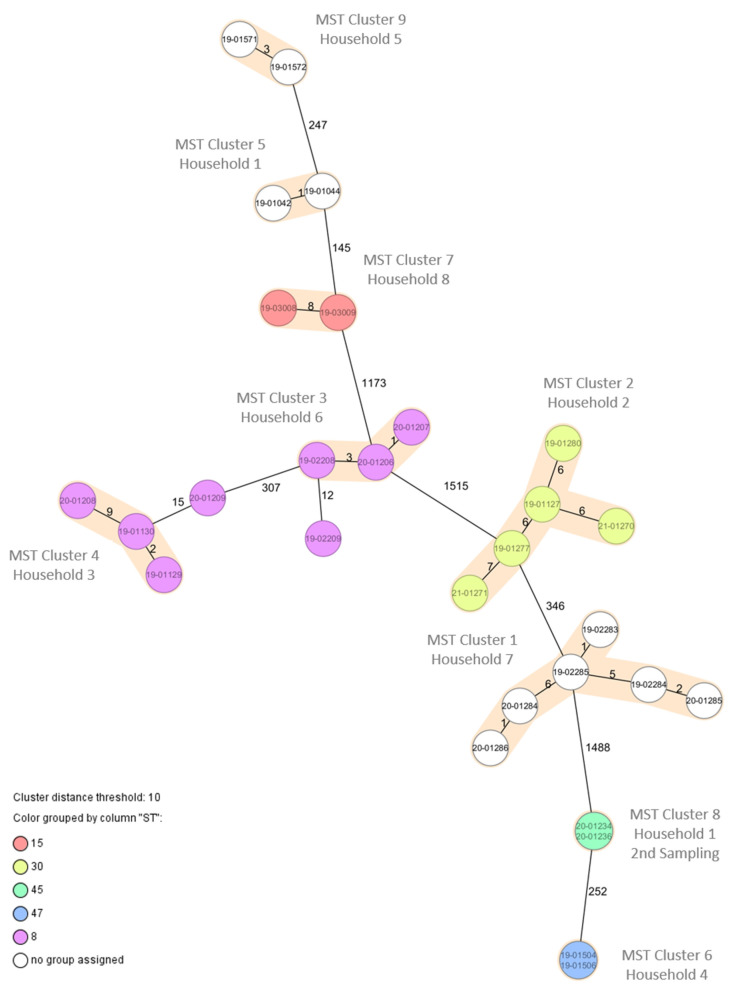
Minimum spanning tree based on core-genome multilocus sequence typing allelic profiles of 27 *S. aureus* isolates from humans and dogs in eight households. Colors indicate the MLST CC (as deduced from WGS data). Brown shades indicate clusters with <10 allelic differences between the isolates.

The overall rate of *S. aureus* carriage among dogs was 10/112 (8.9%), i.e., dogs carrying *S.* *aureus* were found in 10/84 households (11.9%). However, of the ten dogs, eight lived in families where humans were also colonized with *S. aureus*. Among these eight households, there were three with two colonized persons. For all of these households, the isolates of the dogs exhibited the same *spa*-types and, with one exception, the same resistance phenotypes as those isolates derived from the humans living in the same household (Table 3), suggesting intrahousehold transmission between humans and dogs. This was confirmed by the results from the cgMLST allelic profiles derived from whole-genome sequencing of the 27 isolates (Figure 1). Although we observed 1–15 allelic differences between the isolates, this still indicates very close relatedness and transmission [37]. As one transmission was likely for each of the eight households, the transmission rate was 8/42 (19%) and, thus, was lower than that observed for human-to-human transmission (11/38; *p* = 0.4306). A second sampling was performed for these eight households after one year, in which six households participated again. Besides a lack of detection of *S. aureus* in dogs from household No. 1 and, further, No. 2 and No. 4, the results from first sampling were confirmed (Table 3). Interestingly, in household No. 1, a second new dog (puppy) was acquired in that time period, and the human contact person living in that household lost the previous carriage of *S. aureus* CC15 and changed the nasal colonization to *S. aureus* CC45, closely related to that carried by the new dog (Table 3).

#### 3.1.3. *S. pseudintermedius* Colonization of Dogs and Humans

This species was detected in nasal swabs from 42 (37.5%) of the 112 dogs who lived in 36 (42.8%) of the 84 households enrolled in the study. It was obtained from only 1 of the 179 humans (0.6%). Three of these dogs were concomitantly colonized with *S. aureus*. There was no association of *S. pseudintermedius* carriage and the dogs’ hospital stays prior to sampling. Canine *S. pseudintermedius* carriage was not significantly associated with the number of dogs living in the same households (*p* 0.557, Table 4).

**Figure 2:**
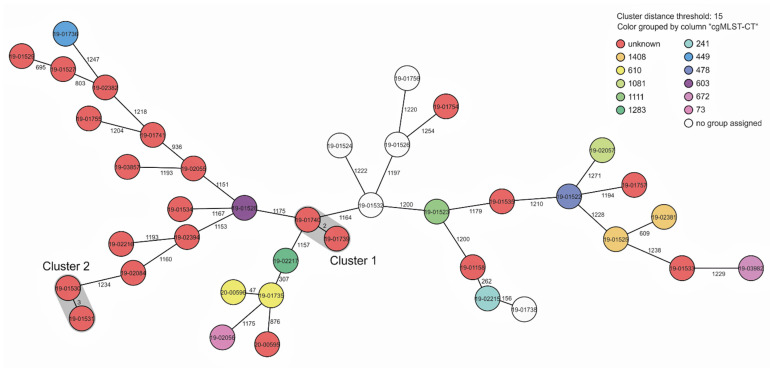
Minimum spanning tree based on core-genome multilocus sequence typing allelic profiles of 39 *S.* *pseudintermedius* isolates from humans and dogs in households (cohort I). Colors indicate the cgMLST-CT (as deduced from WGS data). Grey shades indicate clusters with <15 allelic differences between in the isolates.

In only 1 of 22 households owning two dogs, *S. pseudintermedius* was detected in both dogs. These isolates were closely related when subjected to cgMLST, as shown in Figure 2 (cluster 1: two alleles different). In one of the three households owning three dogs, *S. pseudintermedius* was isolated from all three animals, and in one household owning four dogs, three carried *S. pseudintermedius*, respectively. In these cases, cgMLST typing revealed no relatedness. In only one household owning three dogs, *S. pseudintermedius* was detected in two of the three dogs and one of the three humans belonging to the household. The isolates from the human and from dog 1 were closely related (cluster 2; three alleles different), whereas the isolate from dog 2 was clearly not related. Altogether, the population structure of the sample of isolates investigated was highly diverse, as also indicated by attribution of the isolates to at least 12 complex types (Figure 2). The antibiotic resistance phenotypes of the 42 *S. pseudintermedius* isolates are shown in Table 5. Of these *S. pseudintermedius* isolates, 28 (68%) were susceptible to all tested antibiotics, 8 exhibited resistance to only one antibiotic, and 7 isolates were resistant to two or more antibiotics. One isolate (MRSP) was resistant to oxacillin (mecA positive), penicillin, erythromycin, clindamycin, tetracycline, and gentamicin. This isolate exhibited MLST ST672, as deduced from WGS data.

### 3.2. Cohort II: Dogs and Humans after Visiting a Veterinarian Practice

This group was composed of 17 pairs of dogs and their dedicated owners. Nasal *S. aureus* colonization was detected in 3 of the 17 humans and in 2 of the 17 dogs, which were not dedicated to the human carriers. *S. pseudintermedius* was obtained from six of the dogs, but none of the humans. Of these isolates, three were revealed as MRSP (Table 5).

### 3.3. Cohort III: Dogs Active in AAI and Their Owners

*S. aureus* colonization was detected in 17 (28.3%) of the 60 dog handlers, but in only 1 of the 59 dogs (1.7%) attended by them. The *S. aureus* isolates were attributed to MLST clonal complexes CC5 (2), CC7 (1), CC15 (3), CC22 (3), CC30 (1), CC34 (1), CC45 (4), and CC398 (2). Among these isolates, four were susceptible to all antibiotics tested; four were resistant to penicillin; one was resistant to erythromycin; and one was resistant to penicillin and to fusidic acid. 

*S. pseudintermedius* was detected in seven of the dogs (11.9%) and in one of the human owners (1.7%), whose dog did not carry *S. pseudintermedius*. The isolate from the human was multi-resistant (PEN, OXA, FOS, GEN, ERY, CLI, CIP, MOX, TRS, FUS) and *mecA*- positive (MRSP).

### 3.4. Results from Spa-Typing and Phenotypic Susceptibility Testing of S. aureus (Cohorts I–III)

**Figure 3:**
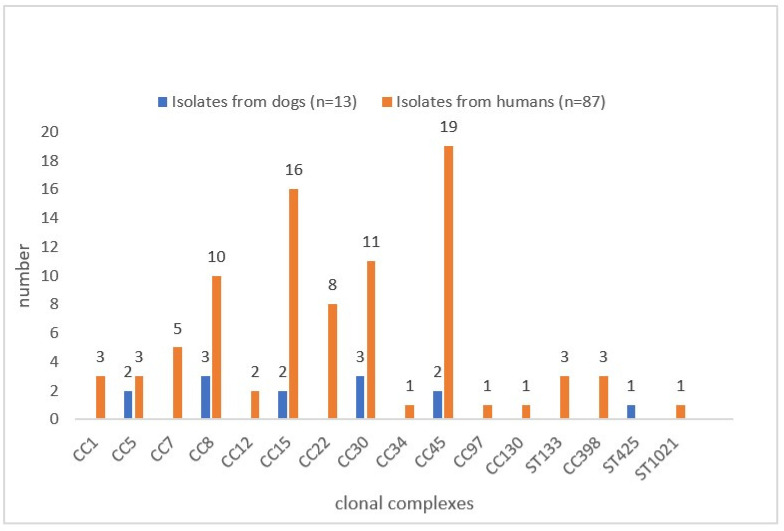
Distribution of MLST clonal complexes (CC) and sequence types (ST) of *S. aureus* isolates from humans and dogs from all cohorts.

The results from *spa*-typing of the 87 isolates from humans are presented in supplemental Table S2. The attribution to clonal complexes of these isolates and of the 13 isolates from dogs is shown in Figure 3. Four clonal complexes predominate in this order: CC45 (*n* = 22), CC15 (*n* = 18), CC30 (*n* = 15), and CC8 (*n* = 13), demonstrated in Figure 3. The results from phenotypical antibiotic susceptibility testing of all detected isolates from the humans and dogs are compiled in supplemental Table S3.

## 4. Discussion

*S. aureus* nasal carriage was observed in 36.9% of the humans living in 42 of 84 households owning dogs. This is more frequent than what has been observed in three previous studies in Central and Northern Germany (26.9% [38], 22% [39]), but in the range of data from other studies, indicating a prevalence of (intermittent) *S. aureus* carriage of 20–50% [2]. Among the factors that predispose a person for *S. aureus* carriage, such as diabetes mellitus or skin disorders/allergies, only atopic eczema was associated with *S. aureus* carriage in this study, which might have been due to the small sample size. The possible influence of living together with dogs on the nasal microbiome [40] and the consequences on *S. aureus* colonization resulting therefrom remain to be explored. Among the 42 households with *S. aureus* carriage in humans, there were 17 in which two and more household members were colonized. Eleven of these isolates from different persons exhibited identical typing patterns, suggesting intrafamilial transmission between humans. This is well known and most probably mediated by direct contact and surface contamination in the household [41,42,43]. On the other hand, this means that, in 55.2% (21/38) of the non-single households with human carriers of *S. aureus* where one member was carrying *S. aureus*, the other human household members were not affected. We observed concomitant carriage of humans and dogs with closely related *S. aureus* isolates in eight households, which suggests transmission between humans and dogs (or vice versa). As it was observed in only 8 among the 42 households (19%) with human *S. aureus* carriers, it seems that transmission between humans and dogs occurs less frequently when compared to intrahousehold human-to-human transmission (39.529.0%, p=0.4306), although this effect was not statistically significant. Similar observations were reported from studies in Spain [44] and the USA [23,45]. Compared with our results, where the typing patterns of *S. aureus* from dogs and humans were identical and clearly indicated transmission, these studies partly found a higher proportion of different isolates. This might have been due to incomplete screening of all the humans in these households. The frequency distribution of clonal complexes to which the MSSA isolates from humans and dogs in our study were attributed corresponded to that observed in a previous study on nasal *S. aureus* colonization in Central Germany [36,39]. So far, there are no indications of dog-adapted clonal lineages of *S. aureus*. The results from a study on the dynamics of *S. aureus* colonization in humans and dogs in seven Spanish households [44] suggested humans as the original reservoir of *S. aureus* for dogs. Our results may support this hypothesis, as the overall carriage rates were much higher among humans than among dogs. However, transmission from dogs to humans also seems to be possible, as suggested by our observation that in one participant, a previously colonizing strain was displaced by another strain, which was also found in a dog that was bought shortly before the sampling. We detected MRSA in 2 of the 179 humans (1.1%), but not in the dogs. This prevalence in humans in the community corresponds to results from the previous studies mentioned above. One of these MRSA belonged to MLST clonal complex CC22, representing the majority of HA-MRSA isolates in Germany [46]. The other MRSA was associated with *mec*C and attributed to clonal lineage ST130, which has a wide range of animal hosts and is rare in humans so far [47]. Dogs acquire MRSA colonization by either animal hospital stay or by contact with humans with MRSA infection or colonization [26,28]; the canine carriage rates in German veterinary practices have previously been assessed and were 2.6%. This is higher than observed in this study. However, the sample size in the comparable cohort (cohort II) of this study was very small. We observed a lower prevalence of nasal *S. aureus* carriage in the humans handling therapy dogs (cohort III) vs. the humans living with dogs in their households (cohort I). An explanation could be that handlers of therapy dogs practice more careful hygiene, including hand washing, while at home or on duty with their dogs. Hands seem to play an important role in human-to-human transmission via the contamination of surfaces [43]. Another explanation may be that the contact is less intense. The prevalence of *S. pseudintermedius* carriage among dogs living in households was higher in our study (37.5%) than that reported for dogs from Spain (23%, [44]), but lower than observed in Canada (46%, [27]) and in Korea (66%, [16]). For the latter study, this could be due to the inclusion of rectal swabs, which might have increased the sensitivity of the analysis. In the study reported from Korea [16], the dogs were sampled at admission to veterinary hospitals. Interestingly, we observed no statistically significant association of the number of dogs living in the 36 households owning colonized dogs with the prevalence of nasal *S. pseudintermedius* colonization. This finding suggests that the acquisition of nasal colonization by transfer between healthy dogs probably does not frequently occur. This is in line with the observed highly diverse population structure, which was also reported for methicillin-susceptible *S. pseudintermedius* from France [48]. We observed nasal carriage of humans living with dogs in only 0.6% of the persons investigated. This is lower than reported from Korea [16], Spain [44], and Canada [27]. It seems that humans are not regular hosts for *S. pseudintermedius*. However, adaptation to humans may occur. A recent pilot study has shown diversity between the isolates attributed to the same clonal lineage from infections in humans and in dogs with respect to pathogenicity islands and virulence gene- containing prophages [49]. In this study, methicillin-resistant *S. pseudintermedius* (MRSP) was only detected in 0.9% of the dogs from households. This might have been due to the low number of dogs with a hospital stay prior to the study. The MRSP isolate exhibited MLST type ST610, which has not been reported so far for MRSP from dogs [12]. The prevalence of MRSP carriage by dogs in the community reported so far is 0.0% (Sweden, [14]), 2.6% (Norway, [50]), and 4.5% (Canada [27]). This is contrasted to the high prevalence observed in dogs undergoing clinical treatment [12,51]. Multi-resistant *S. pseudintermedius* seems to be associated with particular, animal-hospital-associated *S. pseudintermedius* clonal lineages [52], whereas isolates, which are susceptible or resistant to only a few antibiotics, represent natural colonizers of dog. Nevertheless, clinical microbiological laboratories should pay attention to the correct identification of *S. pseudintermedius*, which may be a more common human pathogen than has been recognized so far [53]. Although there is consensus that the benefits of AAI outweigh the risks with respect to the transmission of pathogens [29,54], the question of whether dogs used in AAI may be vectors of MSSA, MRSA, or MRSP remains of interest. Besides older observations [29,30], a current study in a pediatric hospital in the USA has shown mutual transmission of MRSA between dogs and children and the effectiveness of a decolonization procedure on dogs [55]. Guidelines for therapy animal organizations, facilities, and therapy animal handlers providing AAI in healthcare facilities were developed in several countries. SHEA (Society for Healthcare Epidemiology of America) has provided rather detailed guidance [56]. It recommends annual veterinary inspection of the dogs, but not routine screening for MRSA colonization. In contrast, the latter is recommended by the guideline from the German Society for Hospital Hygiene [57]. If the screening of dogs is required (e.g., for outbreak investigations), their handlers should also be screened, as mutual transmission cannot be excluded. So far, there is limited experience with the effectiveness of MRSA (or MSSA or MRSP) decolonization therapies for dogs, which may include the application of a mupirocin nasal ointment. In this context, the finding of high-level mupirocin resistance in MRSA from dogs must be taken into account [58,59]. With respect to alternatives, there is also a single report on the successful application of chlorhexidine [55].

## 5. Conclusions

We estimate the risk of acquisition of nasal colonization of humans with *S. aureus* from colonized dogs in households to be smaller than human-to-human transmission. For *S. aureus*, it seems to be higher than for *S. pseudintermedius*. Whether, in both species, clonal lineages with a more pronounced capacity for spreading among humans and dogs will emerge should be followed up through further surveillance. 

## Figures and Tables

**Table 1:** Characteristics of humans and *S. aureus* (SA) carriage in 84 households.

Characteristics	All 179 Persons in 84 Households ^1^		14 Persons in 8 Households with *S. aureus* in Humans and Dogs		97 Persons in 34 Households with *S. aureus* only in Humans		68 Persons in 42 Households without *S. aureus*
carriage	SA positive	SA negative	SA positive	SA negative	SA positive	SA negative	SA negative
	No. and % among carriers and non-carriers, respectively						
all	66 (36.9)	113 (63.1)	12	2	54 (55.6)	43 (44.3)	68 (100.0)
female	34 (51.5)	61 (54.0)	8	1	26 (48.1)	25 (58.1)	35 (51.4)
male	32 (48.5)	52 (46.0)	4	1	28 (51.9)	18 (41.9)	33 (48.5)
previous hospital stay ^2^	11 (16.7)	15 (13.3)	3	2	8 (14.8)	4 (9.3)	9 (13.2)
antibiotic consumption ^2^	12 (18.2)	12 (10.6)	3	0	9 (16.7)	4 (9.3)	8 (11.8)
diabetes mellitus	4 (6.1)	3 (2.7)	0	0	4 (7.4)	0	3 (4.4)
skin lesions	2 (3.0)	2 (1.8)	0	0	2 3.7)	2 (4.7)	0
atopic eczema	10 (15.2)	4 (3.5%)	1	0	0	0	4 (5.9)
≥2 dogs in the household	25 (37.8%)	36 (31.8%)	5	3	20 (37)	16 (37.2%)	17 (25%)

^1^*p*-values (Fishers exact, two-tailed) for all *S. aureus* carriers vs. non-carriers and characteristics predisposing to *S. aureus* nasal carriage: female gender: 0.759; previous hospital stay: 0.149; previous antibiotic consumption: 0.368; skin lesions: 1.0; diabetes mellitus: 0.426; atopic eczema: 0.02; living with ≥2 dogs in the household: 0.65. ^2^ 6 months prior to sampling.

**Table 2:** Households, number of persons, and *S. aureus* colonization of humans.

Persons/ Household	No. of Households (*n* = 84)	Households with Carriers of *S. aureus* (*n* =42)	Households with 1 Person Colonized			Households with ≥ 2 Persons Colonized		
			Households	Carrier (%)	All persons	Households	Carrier (%)	All persons
1	18	4	4	4 (22)	18			
≥2	66	38 ^1^	21	21 (50)	42	17	41 (82) ^2^	51

*p*-values (Fishers exact, two-tailed): ^1^*S. aureus* carriage in single vs. non-single households, 0.0151 ^2^ *S. aureus* carriage in households with one colonized person vs. households ≥ two colonized persons, 0.0037.

**Table 3:** Demonstration of *S. aureus* in humans und dogs living in the same households.

First Sampling							Second Sampling					
Household	Individual ^1^	Species ^2^	Isolate ^3^	*Spa*-Type	CC ^4^	Resistance Phenotype ^5^	Individual ^1^	Species ^2^	Isolate ^3^	*Spa*-Type	CC ^4^	Resistance Phenotype ^5^
1	H-28	SA	19-01042	t2696	15	PEN	H-28.1	SA	20-01234	t779	45	susceptible
	H-29	SA		t091	7	susceptible	H-29.1	SA		t091	7	susceptible
	D-30	SA	19-01044	t2696	15	susceptible	D-30.1	negative				
							D-400	SA	20-01236	t779	45	susceptible
2	H-62	SA	19-01277	t1577	30	PEN, ERY	H-62.1	SA	21-01270	t1577	30	PEN, ERY
	H-63	SA	19-01127	t1577	30	PEN, ERY	H-63.1	SA	21-01271	t1577	30	PEN, ERY
	D-64	SP				PEN, OXA, TET, CIP, MOX	D-64.1	negative				
	D-65	SA	19-01280	t1577	30	PEN, ERY	D-65.1	negative				
3	H-66	SA	19-01129	t9325	8	PEN	H-66.1	SA	20-01208	t9325	8	PEN
	D-67	SA	19-01130	t9325	8	PEN	D-67.1	SA, SP	20-01209	t9325	8	PEN
4	H-176	SA	19-01504	t026	45	susceptible	H-176.1	negative				
	D-178	SP					D-178.1	negative				
	D-179	SA	19-01506	t026	45	susceptible	D-179.1	negative				
5	H-206	SA	19-01571	t084	15	PEN	H-206.1	No feedback				
	H-207	negative					H-207.1					
	D-208	SA	19-01572	t084	15	PEN						
6	H-312	SA	19-02208	t008	8	PEN, FUS	D-312.1	SA	20-01206	t008	8	PEN, FUS
	D-313	SA	19-02209	t008	8	PEN, FUS	D-313.1	SA	20-01207	t008	8	PEN, FUS
	D-314	SP				susceptible	deceased					
7	H-318	SA	19-02283	t6997	30	PEN	H-318.1	SA	20-01284	t6997	30	PEN
	H-319	SA	19-02284	t6997	30	PEN	H-319.1	SA	20-01286	t6997	30	PEN
	D-320	SA	19-02285	t6997	30	PEN	D-320.1	SA		t6997	30	PEN
8	H-350	negative						No feedback				
	H-351	SA	19-03008	t935	15	susceptible	H-351.1					
	D-352	SP				TET	D-352.1					
	D-353	SA	19-03009	t935	15	susceptible	D-353.1					

^1^ Individual: H = human, D = dog; ^2^ Species: SA = *S. aureus*, SP = **S. pseudintermedius*;* ^3^ laboratory number; ^4^ CC = clonal complex; ^5^ No resistance was detected against: FOS, GEN, LIN, CLI, TIG, VAN, TEI, TRS, RIF, MUP, DAP; susceptible = susceptible to all antibiotics tested.

**Table 4:** Number of dogs in households carrying *S. pseudintermedius*.

		Total No. of Households	No. of Households with SP ^1^ Positive Dogs	Total No. of Dogs	No. of SP ^1^ Positive Dogs
All		84	36	112	42 (37.5%)
Households with 1 dog		61	21 (34.4%)	61	21 (34.4%)
Households with ≥2 dogs		23	15 (65.2%) *p* (0.078)	51	21 (41.2%) *p* (0.557)
	2 dogs	19	11	38	14 (36.8%)
	3 dogs	3	3	9	4
	4 dogs	1	1	4	3

^1^ SP = *S.* *pseudintemedius*.

**Table 5:** Antibiotic resistance phenotypes of *S.* *pseudintermedius* from dogs in the cohorts.

Antibiotic Resistance Phenotypes ^1^	All	Cohort I	Cohort II	Cohort III
PEN	8	7	1	
TET	4	4		
PEN, TET	5	5		
ERY, CLI	3	1	1	1
ERY, CLI, TET	3	2		1
PEN, ERY, CLI, TET	3	3		
PEN, GEN, ERY, CLI, TET, CIP	1	1		
PEN, OXA, TET, CIP, MOX	1	1		
PEN, OXA, ERY, CLI, TET, CIP, MOX, TRS	3		3	
PEN, OXA, GEN, ERY, CLI, CIP, MOX, TRS, FUS	1			1
Susceptible to all antibiotics tested	23	18	1	4
Total	55	42	6	7

^1^ No resistance was found against LIN, TIG, VAN, TEI, RIF, MUP, DAP, CEF.

## Data Availability

Not applicable.

## Supplementary Materials

The following supplementary material is available online at https://www.mdpi.com/article/10.3390/microorganisms10040677/s1. Table S1: Results from *spa*-typing of *S. aureus* isolates from humans and dogs; Table S2: Antibiotic resistance phenotypes of *S. aureus* from humans; Table S3: Antibiotic resistance phenotypes of *S. aureus* from humans.
