# Supplementary material for: Colonization of Dogs and Their Owners with Staphylococcus aureus and Staphylococcus pseudintermedius in Households, Veterinary Practices, and Healthcare Facilities
Source: Microorganisms. 2022 Mar 22;10(4):677. doi: 10.3390/microorganisms10040677 (PMC9024920; doi:10.3390/microorganisms10040677)
Supplement: Supplementary file 1 [file microorganisms-10-00677-s001.zip › Supplemental Table S1.pdf]

Table S1: Matching typing characteristics of *S.aureus* from different persons in each of 11 households and thereof derived numbers of transmission

Household	Individual	*spa*-Type	Antibiotic Resistance Phenotype	Persons with *S.aureus* with matching charactersitics	Number of Transmissions [1]
13	H 62	t1577	PEN, ERY	2	1
	H 63	t1577	PEN, ERY		
17	H 76	t209	PEN, ERY	2	1
	H 77	t209	PEN, ERY		
19	H 85	t015	PEN	2	1
	H 86	t065	susceptible		
	H 89	t015	PEN		
24	H 107	t084	susceptible	2	1
	H 108	t084	susceptible		
25	H 110	t2006	susceptible		
	H 111	t2006	susceptible	3	2
	H112	t2006	susceptible		
34	H 157	t160	susceptible	2	1
	H 158	t160	susceptible		
56	H 252	t2045	PEN		
	H 253	t2045	PEN	4	3
	H 254	t2045	PEN		
	H 255	t2045	PEN		
61	H 281	t2778	PEN		
	H 282	t2778	PEN	3	2
	H 283	t2778	PEN		
63	H 292	t008	PEN, FUS	2	1
	H 293	t008	PEN, FUS		
71	H318	t6997	PEN	2	1
	H 319	t6997	PEN		
83	H 367	t19328	susceptible	2	1
	H 368	t19328	susceptible		
				**Sum**	**15**

[1] No. of transmission = No. of persons carrying *S.aureus* with matching typing characteristics - 1
