# Supplementary material for: Colonization of Dogs and Their Owners with Staphylococcus aureus and Staphylococcus pseudintermedius in Households, Veterinary Practices, and Healthcare Facilities
Source: Microorganisms. 2022 Mar 22;10(4):677. doi: 10.3390/microorganisms10040677 (PMC9024920; doi:10.3390/microorganisms10040677)
Supplement: Supplementary file 1 [file microorganisms-10-00677-s001.zip › Supplemental Table S2.pdf]

Supplemental Table S2: *spa*-typing of *S. aureus* isolates from dogs and humans (cohort I-III)

***spa*- types (number of isolates)**	**Attribution to clonal complexes (CC)**	**Number of isolates**	**Frequency (%)**
Isolates from humans		**87**	**100.00%**
t189 (1), t209 (2)	CC1	3	3.45%
t002 (1), t8337 (2)	CC5	3	3.45%
t091 (3), t1867 (1), t2343 (1)	CC7	5	5.75%
t008 (8), t9325 (1), t1319 (1)	CC8	10	11.49%
t160 (2)	CC12	2	2.30%
t084 (8), t346 (2), t368 (1), t935 (1), t1509 (1), t2696 (1), t4735 (1), t19328 (1)	CC15	16	18.39%
t005 (1), t032 (1), t192 (1), t449 (1), t1709 (1), t2006 (3)	CC22	8	9.20%
t021 (2), t122 (1), t789 (1), t1306 (1), t1577 (3), t2509 (1), t6997 (2)	CC30	11	13.79%
t8270 (1)	CC34	1	1.15%
t015 (4), t016 (1), t026 (2), t065 (2), t116 (1), t130 (1), t620 (1), t728 (1), t779 (1), t2045 (4), t2593 (1)	CC45	19	21.84%
t605 (1)	CC97	1	1.15%
t843 (1)	CC130	1	1.15%
t2778 (3)	ST133	3	3.45%
t1451(1), t7880 (1)	CC398	3	3.45%
t5890 (1)	ST1021	1	1.15%
Isolates from dogs		**13**	**100.00%**
t002 (2)	CC5	2	15.38%
t008 (1), t935 (1), t9325 (1)	CC8	3	23.08%
t084 (1), t2696 (1)	CC15	2	15.38%
t1577 (1), t6997 (1), t7880 (1)	CC30	3	23.08%
t026 (1), t779 (1)	CC45	2	15.38%
t19469 (1)	ST425	1	7.69%
