# Supplementary material for: Colonization of Dogs and Their Owners with Staphylococcus aureus and Staphylococcus pseudintermedius in Households, Veterinary Practices, and Healthcare Facilities
Source: Microorganisms. 2022 Mar 22;10(4):677. doi: 10.3390/microorganisms10040677 (PMC9024920; doi:10.3390/microorganisms10040677)
Supplement: Supplementary file 1 [file microorganisms-10-00677-s001.zip › Supplemental Table S3.pdf]

Supplemental Table S3: Antibiotic resistance phenotypes of *S. aureus* from humans

Antibiotic resistance phenotypes	Frequency (%)	Resistance against singular antibiotics*	Frequency (%)
Isolates from humans			
PEN	36 (41.3)	PEN	53 (61.0)
PEN, OXA	1 (1.1)	OXA	2 (2.3)
PEN, OXA, ERY, CLI, CIP, MOX	1 (1.1)	ERY	12 (13.8)
PEN, GEN, ERY, CLI, CIP, MOX	1 (1.1)	CLI	2 (2.3)
PEN, GEN, ERY	1 (1.1)	GEN	2 (2.3)
PEN, ERY	7 (8.0)	TET	1 (1.1)
PEN, FUS	5 (5.8)	CIP	2 (2.3)
PEN, TET	1 (1.1)	MOX	2 (2.3)
ERY	2 (2.3)	FUS	5 (5.8)
Suceptible	32 (36.8)		
**Sum**	**87**		
Isolates from dogs			
PEN	3	PEN	5
PEN, ERY	1	ERY	1
PEN, FUS	1	FUS	1
Susceptible	8		
**Sum**	**13**		

*All of the isolates were susceptible to Rifampicin, (RIF), Linezolid (LIN), Daptomycin (DAP),
Trimethoprim-Sulfamethoxazol (SXT), Vancomycin (VAN), Teicoplanin (TEI) und Mupirocin (MUP)

Abbreviations: Penicillin (PEN), Oxacillin (OXA), Fosfomycin (PHO), Gentamicin (GEN), Linezolid (LIN), Erythromycin (ERY), Clindamycin (CLI), Tetracycline (TET), Tigezyklin (TIG), Vancomycin (VAN), Teicoplanin (TEI), Ciprofloxacin (CIP), Moxifloxacin (MOX), Fusidicacid- Sodium (FUS), Rifampicin (RIF), Trimethoprim-Sulfamethoxazol (SXT), Mupirocin (MUP), Cefotaxim (CEF), Daptomycin (DAP)
